# Relationship of Serum Antileishmanial Antibody With Development of Visceral Leishmaniasis, Post-kala-azar Dermal Leishmaniasis and Visceral Leishmaniasis Relapse

**DOI:** 10.3389/fmicb.2019.02268

**Published:** 2019-10-09

**Authors:** Dinesh Mondal, Prakash Ghosh, Rajashree Chowdhury, Christine Halleux, Jose A. Ruiz-Postigo, Abdul Alim, Faria Hossain, Md Anik Ashfaq Khan, Rupen Nath, Malcolm S. Duthie, Axel Kroeger, Greg Matlashewski, Daniel Argaw, Piero Olliaro

**Affiliations:** ^1^Emerging Infections and Parasitology Laboratory, International Centre for Diarrhoeal Disease Research, Dhaka, Bangladesh; ^2^UNICEF/UNDP/World Bank/WHO Special Programme for Research and Training in Tropical Diseases (TDR), World Health Organization, Geneva, Switzerland; ^3^Department of Neglected Tropical Diseases, World Health Organization, Geneva, Switzerland; ^4^Infectious Disease Research Institute, Seattle, WA, United States; ^5^Centre for Medicine and Society, University Medical Center Freiburg, Freiburg im Breisgau, Germany; ^6^Department of Microbiology and Immunology, McGill University, Montreal, QC, Canada; ^7^Centre for Tropical Medicine and Global Health, Nuffield Department of Medicine, University of Oxford, Oxford, United Kingdom

**Keywords:** visceral leishmaniasis (VL), treatment failure (TF), relapse VL (RVL), Post-kala-azar dermal leishmaniasis (PKDL), rK39 antibody, ELISA, predictive biomarker

## Abstract

**Introduction:**

To sustain the achievement of kala-azar elimination program (KEP), early detection and treatment of the visceral leishmaniasis (VL) cases and associated modalities such as treatment failure (TF), relapse VL (RVL), and Post-kala-azar dermal leishmaniasis (PKDL) is the cornerstone. A predictive biomarker for VL development and related complications could also play a crucial role in curtailing disease incidence and transmission. Investigations to find a biomarker with prospective capabilities are, however, scarce. Using samples and known clinical outcomes generated within two previous longitudinal cohort studies, we aimed to determine if fluctuations in serum anti-rK39 antibody levels could provide such predictive value.

**Materials and Methods:**

Serum samples collected at four different time points (Baseline, 12, 18, and 24 months) from 16 patients who had developed VL within the monitoring period and 15 of their asymptomatic healthy controls counterparts were investigated. To investigate potential prediction of VL related complications, serum samples of 32 PKDL, 10 RVL, 07 TF, and 38 cured VL from a single dose AmBisome trial were analyzed. Of this second panel, all patients were monitored for 5 years and sera were collected at four time points (Baseline then 1, 6, and 12 months after treatment). The level of anti-rK39 antibodies in archived samples was measured by a semi-quantitative ELISA.

**Results:**

The mean antibody level was significantly higher in VL patients compared to their asymptomatic healthy counterparts at each time point. Likewise, we observed a trend toward elevations in antibody levels for PKDL, RVL, TF relative to the reducing levels observed in cured VL. Receiver operating characteristic (ROC) analysis found a promising predictive power of rK39 antibody levels to reveal progression from asymptomatic *Leishmania donovani* infection stage to VL, defined as 87.5% sensitive and 95% specific. Following treatment, rk39 antibody notably showed 100% sensitivity and 95% specificity in predicting TF.

**Conclusion:**

Our data indicate that the relative quantity of serum anti-rK39 antibody has promise within either a predictive or prognostic algorithm for VL and VL-related modalities. These could enable VL control programs to implement more effective measures to eliminate the disease. Further research is, however, imperative to standardize the rK39 antibody ELISA between sites prior to broader use.

## Introduction

Visceral leishmaniasis (VL) is one of the twenty neglected tropical infectious diseases (NTDs) listed by the World Health Organization (WHO). On the Indian sub-continent, where VL is known also as kala-azar, it is caused by *Leishmania donovani* (LD) transmitted during the blood meals of female *Phlebotomus argentipes* sand flies (Chappuis et al., 2007). The first reported outbreak of VL was in 1824 in the Jessore of British Bengal and killed 75,000 people within 3 years (Bern and Chowdhury, 2006). Since then, periodic epidemics of VL have been common on the Indian sub-continent. Besides, 90% of VL cases occur in mainly six countries including Bangladesh, India, Nepal, Ethiopia, Sudan, and Brazil (Chappuis et al., 2007).

For VL case management, control and elimination, critical remaining questions are which individuals are at risk of becoming diseased and who can transmit the infection. A person may have several outcomes after being infected by *L. donovani*. Approximately 90% of infected individuals do not develop VL symptoms but rather remain asymptomatic (dos Santos et al., 2016), while about 10% go on to develop clinically overt disease with fever, enlarged spleen, anemia or pancytopenia (VL patient) (unpublished data). While VL is almost invariably fatal if left untreated, timely and proper treatment with current therapies is mostly effective, with only 2–3% of cases failing to respond to initial treatment (treatment failure, TF) (Mondal et al., 2014). A recent cohort study from Bangladesh established that 3–7% of initially cured VL patients may develop VL again, and this is collectively defined as VL relapse (VLR) (Mondal et al., 2019). In Bangladesh, 10–20% of cured VL patients may develop Post-kala-azar dermal leishmaniasis (PKDL), a dermatological sequela of VL, usually within 3 years following treatment (Mondal et al., 2019). The skin lesions of PKDL can be macular, papular, nodular, and polymorphic (Zijlstra et al., 2003; Mukhopadhyay et al., 2014). In Bangladesh approximately 10% of asymptomatic may also develop PKDL without having had clinical VL (Rahman et al., 2010).

While VL patients are generally believed to be the engine of *L. donovani* transmission, we have demonstrated that VL, VLR, and PKDL cases can all transmit LD to sand flies (Addy and Nandy, 1992; Molina et al., 2017; Mondal et al., 2018). The transmission potential of a VL and PKDL case through a single sand fly bite is nearly 80 and 65%, respectively (Mondal et al., 2018). In reality, however, this theoretical potential may be altered by factors such as frequency of exposure to bites and healthcare seeking behaviors. Being symptomatic pushes VL cases to seek medical care actively, though often they experience delays of several months’ times before receiving proper treatment, a period during which they continue to be a source of infection (Mondal et al., 2009). Conversely, PKDL cases usually do not seek medical care until they are stigmatized by their skin lesions and they therefore can remain a source of transmission for years (Basher et al., 2015). Theoretically, asymptomatic infections could also be transmitters of the infection, especially in the pre-patent period, but this has not yet been formally documented.

Under the aegis of the WHO, the Governments of Bangladesh, India, and Nepal committed to reduce and maintain the incidence of VL less than 1 per 10,000 people at the upazila (sub-district), block and district level respectively, to eliminate VL as a public health problem (World Health and Organization, 2005). While the kala-azar elimination program (KEP) has achieved remarkable successes in reducing VL incidence throughout the Indian subcontinent, it will not be possible to sustain elimination unless sources of new infections are detected and managed early. Much research has been conducted in an effort to detect *L. donovani* infection and VL-associated complications (Chappuis et al., 2007). As a means to provide early detection of relapsing VL, Mollet et al. (2018) conducted a case-control study and reported a significant difference in rK39 IgG1 level between cured and relapse VL (RVL) cases. A cohort study, however, has not yet been conducted to validate the findings Moreover, very few prospective cohort studies have been conducted to enable the prediction of VL and VL-associated modalities such as TF, RVL, and PKDL (Gasim et al., 2000; Hasker et al., 2014; Chapman et al., 2015; Chakravarty et al., 2019). Therefore, evaluation of new or available biomarkers that can potentially predict VL and its associated modalities are still required. Such studies, and the biomarkers they may reveal, could have a crucial role in facilitating control strategies in consolidation and maintenance phases of the KEP.

To address the current gaps in prognosis of VL and VL-associated modalities we investigated the dynamics of anti-rK39 antibodies by longitudinal comparative assessment in asymptomatic and emergent VL patients to determine if altered levels can predict those most likely to develop VL. Among a second serum panel, we evaluated if quantitative measurement of anti-rK39 antibodies could reveal VL cases at risk for development of PKDL, TF or relapse.

## Materials and Methods

### Study Design and Site

The study was a laboratory-based evaluation and was carried out in International Centre For Diarrhoeal Disease Research, Bangladesh (ICDDR,B), Dhaka, Bangladesh from November 2015 to November 2017 using archive samples, patients’ metadata and informed voluntary consent from adult patients and guardian of minor patients of previous studies.

### Study Population, Sampling Method, and Sample Size

#### Asymptomatic Subjects Included in This Study

**FIGURE 1:**
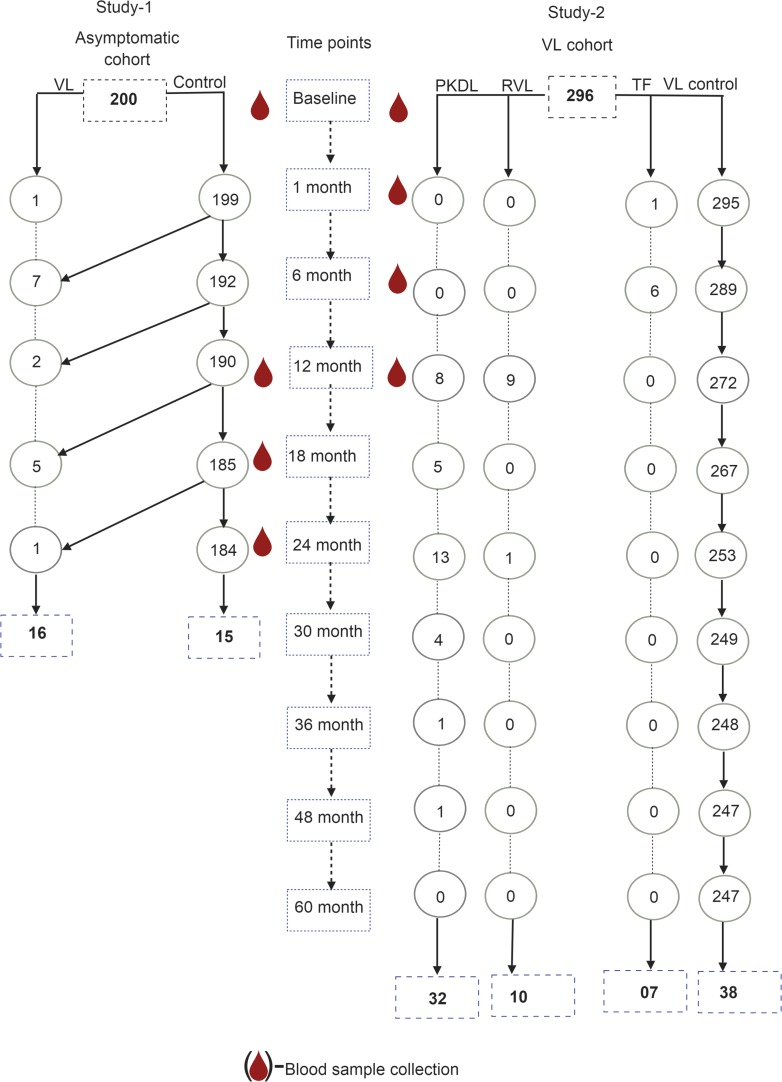
Flow chart for selection of study subjects from asymptomatic and VL cohorts.

The asymptomatic cohort included 200 individuals from a trial involving micronutrient intervention and de-worming to prevent the development of symptomatic VL (trial registration number NCT01069198). These were apparently healthy asymptomatic individuals living in a VL-endemic village, without VL or PKDL symptoms but positive on the rK39-antibody detection rapid test. Children aged less than 2 years, adults aged more than 60 years, pregnant women and persons with signs and symptoms of any chronic disease were not enrolled, nor were non-consenting individuals. The study ended on October 30, 2012 and 196 of 200 completed (98%) the 2-year follow-up. Serum samples were analyzed at baseline, 12, 18, and 24 months from the 16 subjects who developed symptomatic VL and were treated following national guidelines in the public hospital in Trishal, Mymensingh, as well as those of 15 asymptomatic controls (rK39-positive, asymptomatic subjects without subsequent VL) (Figure 1).

#### VL Patients Included in This Study

The study included serum samples from VL patients (before and after treatment) of a previous study (PR-11001 and amendment SDA–Trial registration number ACTRN12612000367842). This study was originally a single-arm clinical trial for VL patients who were treated with 10 mg/kg body weight single-dose liposomal Amphotericin B (LAmB). The study started in February 2012 and was completed in August 2013, but was subsequently amended to allow for a 5-years follow-up of the cohort (VL cohort), which was completed in 2016. A VL patient was defined according to the National Kala-azar Elimination Guideline as a person living in the kala-azar endemic area with fever lasting for more than 2 weeks, splenomegaly and a positive rK39 rapid test (Ahmed et al., 2014). The study enrolled 300 VL patients with the following entry criteria and 296 patients completed study per protocol.

##### Inclusion criteria

Both sexes, age ≥5 years, history of fever for more than 2 weeks, splenomegaly, positive rK39 rapid test, hemoglobin ≥5 g/dl, written informed consent from the patient, or parent or guardian if under 18 years old.

##### Exclusion criteria

History of intercurrent or presence of clinical signs/symptoms of concurrent diseases/conditions (e.g., chronic alcohol consumption or drug addiction, renal, hepatic, cardiovascular or CNS disease; diabetes mellitus, dehydration, other infectious diseases or major psychiatric diseases) only if the intercurrent conditions were not under control before starting LAmB treatment; any condition which, according to the investigator, might prevent the patient from completing the study therapy and subsequent follow-up; history of allergy or hypersensitivity to Amphotericin B; previous treatment for VL within 2 months of enrollment into the study; prior TF with Amphotericin B; PKDL; pregnancy.

Seven and 289 of 296 had respectively TF and final cure by day 180 since treatment. Ten and 32 of the 289 cured cases went on to develop respectively VLR and PKDL during the 5-year follow-up. Two hundred forty seven of 289 did not have TF/VLR/PKDL during 5-year follow-up (Figure 1). The definitions of TF, VLR, and PKDL were:

**Treatment failure (TF):** A treated VL patient who had no clinical improvement at 30 days after treatment, or had an initial improvement at 30 days followed by recurrence of sign and symptom of VL and had LD bodies in spleen aspirates within day 180 after treatment.

**VL relapse (VLR):** A VL patient who is cured at the 180-day visit who had reappearance of symptoms and LD bodies in spleen aspirates during subsequent follow-up.

**Post-kala-azar Dermal Leishmaniasis (PKDL):** A cured VL patient at day 180 with no relapse and with skin lesions (macular, popular, nodular or polymorphic), positive on the rK39 rapid test, negative for fungal infection by microscopic examination and negative for leprosy using skin sensation test and with skin specimen positive for LD bodies by microscopy and/or positive for LD DNA by qPCR.

Randomly selected 38 of 247 cured patients served as control (VL control) in this study. TF and VLR were treated with multiple-dose LAmB (a total dose of 15 mg/kg body weight divided into three, 5 mg/kg applications given) every other day. PKDL cases were treated following national guideline (Ahmed et al., 2014).

#### Laboratory Methods

For asymptomatic individuals included in this study, rK39 rapid test and rK39 ELISA were performed on serum samples corresponding to baseline, month 12, month 18, and month 24. For former VL patients, rK39 rapid test and rK39 ELISA were performed on serum samples corresponding to baseline, day 30, day 180, and day 365.

#### Rapid Diagnostic Test

The rK39 rapid diagnostic test (RDT) was performed using serum by Kala-azar detect Rapid test (InBios, Seattle, WA, United States) following the manufacture’s instruction.

#### Semi-Quantitative ELISA

A semi-quantitative rK39 ELISA was performed with improvisation to quantify antibody levels in serum as previously described (Vaish et al., 2012; Ghosh et al., 2016). In brief, flat bottom 96-well microtiter plates (Greiner-Bioone) were coated with 25 ng/well of rK39 antigen in PBS (pH 7.4) and incubated overnight at 4°C. On the following day, plates were washed three times with wash buffer (PBS containing 0.05% Tween 20, pH 7.4) and then blocked with blocking buffer (PBS containing 1% BS, pH 7.4) for 3 h at 37°C. Following 5 times wash with wash buffer, 50 μl of 1:400 diluted serum sample was added to the respective wells and incubated for 1 h at 37°C. After incubation, the plates were washed five times with wash buffer and peroxidase-conjugated rabbit anti-human IgG (Jackson Immune-Research, United States) (1:5000 times dilution in DF) was added to each well and incubated for 1 h at 37°C. After washing five times, 100 μl of TMB substrate (Sigma, United States) was added in each well then the plate was placed in the dark for 20 min. The optical density (OD) was measured at 450 nm in a micro-plate reader. A standard curve was constructed for each plate with serially diluted positive pooled serum and antibody levels were estimated from the standard curve. Pooled serum was serially diluted two times to get a range from 16,000 to 4,096,000 for preparing the standard curve. The antibody level was expressed as arbitrary unit (AU) for each dilution. 1000 AU was assigned to the OD value of the pooled serum corresponding to 16,000 times dilution. Antibody level in experimental serum samples was determined from the standard curve.

### Data Management and Statistical Analysis

After computing lab data from laboratory reporting forms, descriptive and inferential statistics were carried out. Comparisons between means were done by a parametric and non-parametric method when applicable. Proportions between groups were compared by Chi-square test. The receiver operating characteristic curve (ROC) approach was used to find the cutoff for prediction of asymptomatic at risk for VL and VL patients at risk for an unfavorable outcome. A two-tailed *p*-value of ≤0.05 was considered as significant. Data analysis was performed with SPSS version 20.

### Quality Assurance

Serum samples were archived at −20°C immediately after separation from blood and at −80°C for long time archives. Pooled serum samples from confirmed VL patients were aliquoted in multiple tubes to avoid repetitive fridge-thaw cycles during ELISA. Each sample was run in triplicate to measure the appropriate arbitrary titer of antibody.

### Research Ethics

This research was approved by the ICDDR, B, ethics committee (Dhaka, Bangladesh) and the WHO Ethics Review Committee. All study participants or their guardians of previous studies provided written informed consent for use of their collected samples for subsequent studies on VL research.

## Results

### Population Characteristics and Risks for VL, TF, VLR, and PKDL

Of the 200 asymptomatic rK39 antibody-positive subjects who completed the 2-year follow-up, 16 (8.2%) went on to develop symptomatic VL of which 10 (5.1%) developed disease within the first year of follow-up. Of the 296 patients treated for VL who completed the 3-year follow-up, 7 (2.4%) failed treatment by 6 months, and 10 (3.4%) and 32 (10.8%), respectively relapsed or developed PKDL between month 6 and 5 years post-treatment (Table 1).

**TABLE 1:** Population characteristics of asymptomatic participants with rK39-positive and VL patients.

**Cohort**	**Subject type (*n*)**	**Sex % female (*n*)**	**Age in years (95% CI)**	**Body mass index (95% CI)**	**Hemoglobin g/dl (95% CI)**
Asymptomatic	with subsequent VL (16)	44 (7)	14.78 (8.13–21.44)	15.70 (14.34–17.05)	12.53 (11.26–13.80)
	without subsequent VL (15)	47 (7)	15.94 (14.34–17.05)	15.94 (14.40–17.47)	12.80 (11.57–14.11)
	*p*-value between groups	0.56	0.90	0.80	0.71
VL patients	Treatment failure (7)	14.3 (1)	114.9 (5.0–224.7)	13.6 (11.9–15.3)	7.8^#^ (6.5–9.1)
	VL relapse (10)	50.0 (5)	169.2 (74.1–264.3)	14.1 (11.6–16.5)	10.1 (8.8–11.4)
	Post-kala-azar Dermal Leishmaniasis (32)	37.5 (12)	247.1 (184.3–309.9)	15.3 (14.2–16.3)	9.3 (8.7–10.0)
	VL control (38)	34.2 (13)	231.5 (175.9–287.1)	15.2 (14.4–16.0)	10.1^#^ (9.5–10.6)
	*p*-value between groups	0.49	0.21	0.16	0.82

*#*p* = 0.008.*

The study population included 16 VL conversions from 200 asymptomatic cases and 15 asymptomatic cases as the counterpart of VL patients who did not convert to VL and 7, 10, 32 and 38 VL patients, respectively with TF, VLR, PKDL, and VL controls with favorable treatment outcome. Asymptomatic subjects with and without subsequent VL did not differ statistically significantly regarding their sex, body mass index and blood hemoglobin levels at enrollment (Table 1). The TF, VLR, PKDL, and VL control also did not differ significantly regarding their sex, age, and body mass index. However, the blood hemoglobin of TF patients was statistically significantly lower than VL controls (Table 1).

The risk for development of VL within next 24 months after being diagnosed as asymptomatic by the rK39 RDT was 8.2% (Table 2). The risks for TF, VLR and PKDL among VL patients within 5 years post-treatment with single-dose LAmB was respectively 2.4, 3.4, and 10.8% (Table 2).

**TABLE 2:** Risks for VL, TF, VLR, and PKDL.

**Condition**	**Denoted by**	**Probability by month *x***	***n* with condition**	***N* evaluable**	**Risk %**	**95% CI**
Infected asymptomatic	A					
Develop clinical VL	V	P (V¦A)24	16	196	8.2	4.3–12
Do not develop VL	∼V	P (∼V¦A)24	180	196	91.8	88–95.7
VL case treated	Vt					
Fail treatment	F	P (F¦Vt)60	7	296	2.4	0.6–4.1
Relapse post-treatment	R	P (R¦Vt)60	10	296	3.4	1.3–5.4
Develop PKDL	D	P (D¦Vt)60	32	296	10.8	7.3–14.3
Definitive cure	∼V	P (∼V¦Vt)60	247	296	83.4	79.2–87.7

### Dynamic of rK39 Antibody Titer in Asymptomatic *L. donovani*-Infected Individuals

**FIGURE 2:**
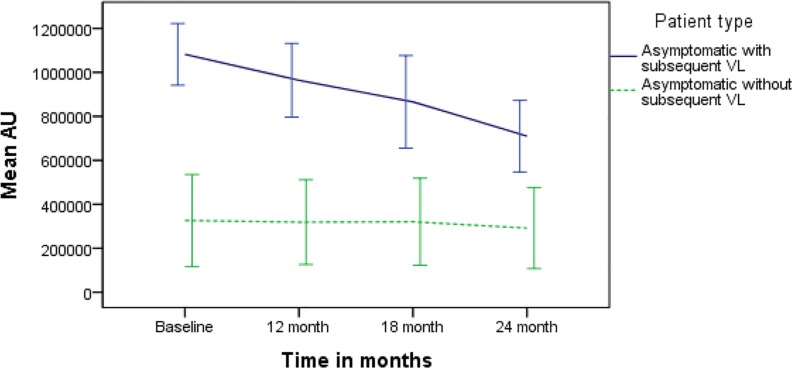
Comparison of serum rK39 titers between asymptomatic with and without VL at baseline, 12, 18, and 24 months.

**FIGURE 3:**
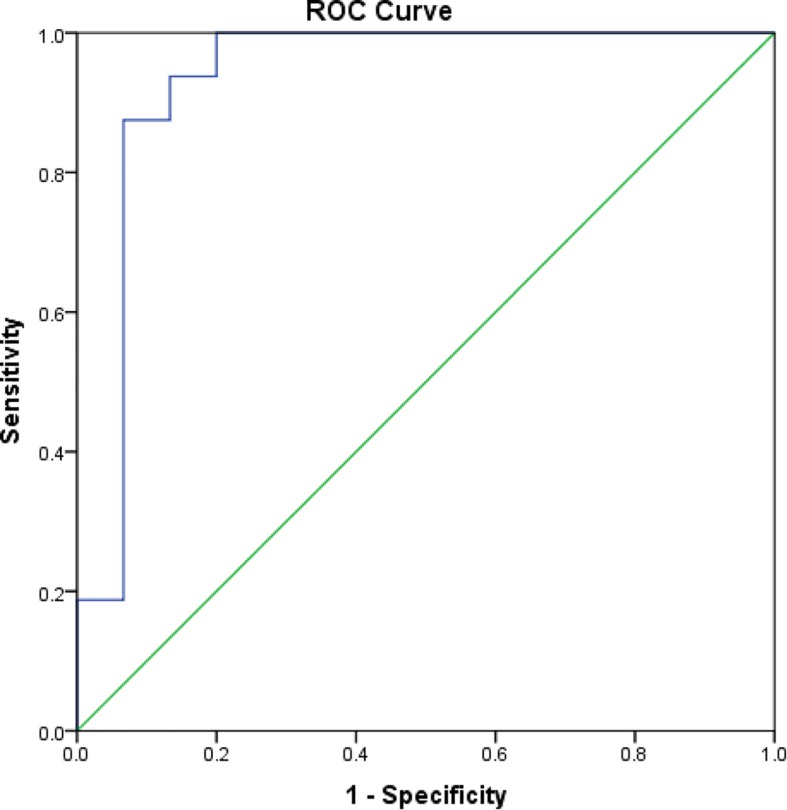
Receiver operating characteristic curve for cutoff of rK39 AB titer for prediction of asymptomatic at risk for VL within subsequent 24 months.

The mean rK39 antibody titers as determined by ELISA from asymptomatic cases who developed VL were significantly higher than those from asymptomatic cases who did not develop VL throughout the 2-year follow-up (Figure 2). The serum titers of rK39 antibodies were inversely related to hemoglobin level (*r* = −0.182, *p* = 0.043) while they did not vary with sex, age, and BMI. The ROC curve shows a cutoff of rK39 antibody titer of 802,497 AUs predicts asymptomatic infection progression to VL within the next 24 months with a sensitivity of 87.5% and specificity of 95% (Figure 3).

We determined the relation between the rK39 RDT result and the rK39 antibody ELISA along with the longevity of the rK39 RDT-positive results. The planned number of rK39 RDT and ELISA evaluations in 31 asymptomatic at 4 time points was 124, of which 116 tests were completed (94%). The average (95% CI) arbitrary rK39 antibody titer for RDT negative (*n* = 20) and RDT positive test (*n* = 96) was respectively 308,986 (95% CI, 1,25,406–4,92,565) and 670,560 (5,78,173–7,62,948), *p*-value = 0.002. All asymptomatic cases had a positive rK39 RDT at baseline as per enrollment criteria. Five of 16 (33%) asymptomatic *L. donovani*-infected individuals that developed VL had a negative rK39 RDT after treatment, however, at 24 months all but one was positive. About 60% (9/15) asymptomatic cases that did not develop VL had negative rK39 RDT at some point during follow-up, but at 24 months only 20% (3/15) had a negative rK39 RDT result (Table 2).

### Trend of rK39 Antibody Titer in VL Patients

**FIGURE 4:**
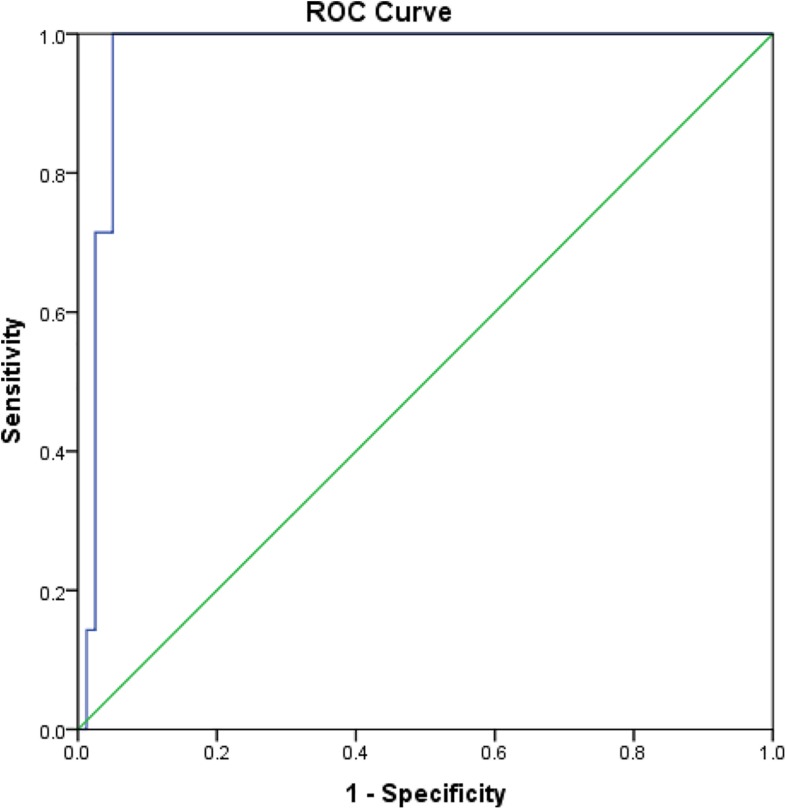
Receiver operating characteristic for prediction of VL patients with subsequent treatment failure.

Comparisons of rK39 antibody titers between TF, VLR, PKDL, and VL control are given in Table 3. The TF group had the lowest titers compared to all other groups at all-time points except at day 365 when it did not differ from control. This indicates that the rK39 antibody titer at day 30 could potentially predict TF. An arbitrary titer of rK39 antibodies ≤626,195 at baseline before treatment predicted TF in this group with a sensitivity and specificity of 100 and 95%, respectively (Figure 4).

**TABLE 3:** Comparison of mean serum rK39AB titer (95% CI) different groups at different time points.

**Time**	**TF (*N* = 7) (95% CI)**	**VLR (*N* = 10) (95% CI)**	**PKDL (*N* = 32) (95% CI)**	**VL control (*N* = 38) (95% CI)**
Baseline	399,461^@^ (306,173–492,749)	878,968^#^ (666,143–1,091,794)	1,558,044 (1,497,948–1,618,139)	1,456,777 (1,323,891–1,589,664)
Day 30	294,483^@^ (154,558–434,408)	1,103,701^&^ (879,257–1,328,145)	1,516,932 (1,433,444–1,600,421)	1,400,044 (1,250,620–1,549,467)
Day 180	426,072^@^ (319,139–533,006)	1,256,668 (858,581–1,654,754)	1,299,201 (1,129,658–1,468,745)	1,039,447 (853,583–1,225,312)
Day 365	619,544^@@^ (224,207–1,014,880)	1,516,307 (1,370,435–1,662,180)	1,370,186^∗∗^ (1,205,538–1,534,834)	935,618 (777,604–1,093,632)

*^@^*p* < 0.01 compared to all other groups; ^#^*p* < 0.001 compared to PKDL and Control; ^&^*p* < 0.001 compared to PKDL; ^@@^*p* = 0.001 compared to VLR & PKDL; ^∗∗^*p* = 0.001 compared to control.*

**FIGURE 5:**
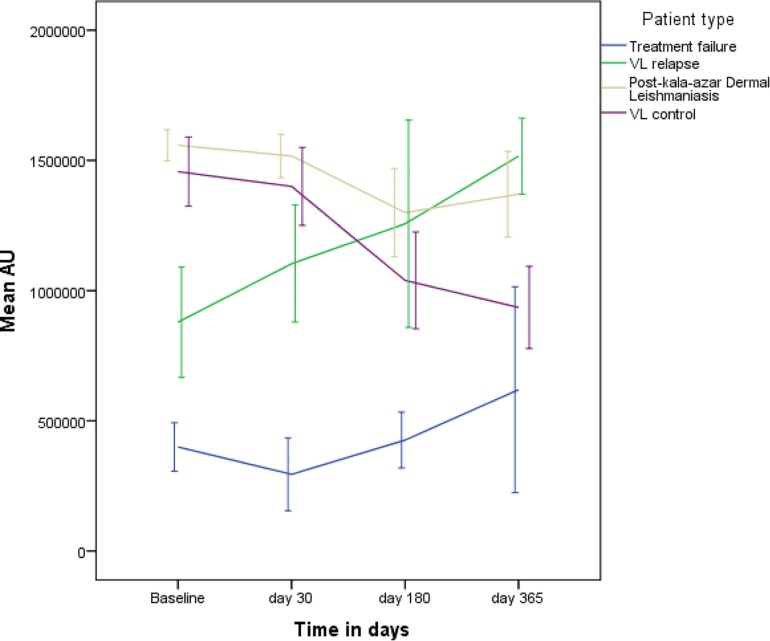
Dynamics of serum rK39AB titer of TF, VLR, PKDL, and VL controls.

**FIGURE 6:**
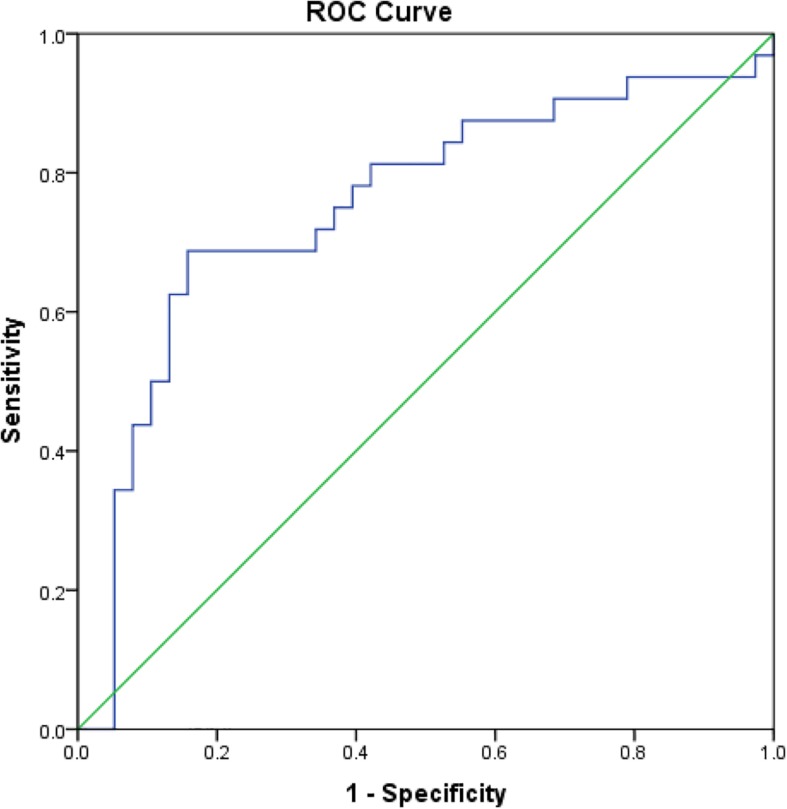
Receiver operating characteristic curve for cutoff of rK39 AB titer at 365 day since treatment for prediction of cured VL at risk for PKDL within subsequent 24 months.

The dynamic of rK39 antibody titers of TF, VLR, PKDL and VL control was different (Figure 5). The rK39 antibody titer of VL controls was not statistically different from the titer of VLR and PKDL at baseline, day 30 and 180 after treatment (Table 3 and Figure 5). At day 365, however, the mean titer of the VL controls was significantly lower than that of PKDL and VLR indicating that the rK39 antibody titer at 1 year could be used to identify cured VL cases at risk for PKDL/VLR (Table 3 and Figure 5). ROC analysis showed that on day 365 an rK39 antibody titer of ≥1,400,000 predicts development of PKDL with a sensitivity of 70% and specificity of 85% (Figure 6). A rK39 antibody titer ≥1,518,584 predicts VLR with a sensitivity and specificity of 60 and 87%, and a titer of ≥1,455,535 predicts PKDL/VLR with a sensitivity and specificity of 67 and 86%, respectively. Regarding the rK39 RDT, all patients remained positive at day 180, while at day 365, 57% (4/7), 100% (10/10), 94% (30/32), and 84% (32/38), respectively with TF, VLR, PKDL, and VL control were still positive by rK39 RDT.

## Discussion

There are several important observations made in this study. High levels of serum antibodies to rK39 could help predict which asymptomatic individuals will progress to VL. Determining antibody levels to rK39 could also predict TFs and relapse in treated VL patients. Interestingly, low serum antibody titers to rK39 were found to be associated with TF. Our study also revealed that the majority of VL patients remained positive by the rK39 RDT for a long time after successful treatment, whereas this was variable in case of asymptomatic *L. donovani* infections. The study also quantifies risks of subjects being potential sources of transmission: about 8% of asymptomatic infections progressed to symptomatic VL, and when treated about 17% of these, either did not respond, relapsed or developed PKDL.

There are several published cohort studies that have assessed risk factors for asymptomatic *L. donovani* infection progressing to VL and long-term risk of treated VL patients for PKDL and relapse (Gasim et al., 2000; Hasker et al., 2014; Chakravarty et al., 2019). A recent study from India found that higher titers in direct agglutination test (DAT) and rK39 evaluations were associated with higher odds for progression to VL (Hasker et al., 2014; Chakravarty et al., 2019). This study corroborates previous findings of a strong association between high rK39 antibody titers and subsequent progression to disease from an asymptomatic state (Hasker et al., 2014; Chakravarty et al., 2019). Furthermore, this study substantiates the previous findings on the prognostic value of rK39 antibody toward development of RVL and/or PKDL following the treatment for VL (Singh et al., 1995, 2002; Houghton et al., 1998; Kumar et al., 2001). This is however, the first study to explore the predictive value of rK39 antibody titers for developing PKDL and VLR after successful VL treatment, and the first to provide evidence of lower rK39 antibody titers associated with VL TF.

Achieving the KEP target of less than 1 VL case per 10,000 inhabitants at the upazila level in Bangladesh may not be enough to ensure long-term sustainability. Evidence collected through this and other studies highlights the risks of continuing transmission and potential resurgence of the disease. Unfortunately, we do not have the right intervention tools to aim for zero transmission. Key to this is the ability to identify early LD-infected individuals who can potentially transmit, including asymptomatic cases likely to develop symptomatic VL, PKDL, and VL treatment relapses. Although there is no treatment regimen for asymptomatic infections, the high antibody titer could serve as a biomarker for periodic follow-up of such latent cases. Mathematical models show that early diagnosis of VL is important to eliminate the disease in line with the WHO recommendations for prompt effective treatment to curtail transmission and improve treatment outcome (WHO, 2010; Medley et al., 2015; Welay et al., 2017; Le Rutte et al., 2018). An individual’s poor prognosis is sometimes linked to other variables such as age or low platelet count (Braga et al., 2013). Our study findings could provide valuable direction to adapt such mathematical models to explore the importance of early diagnosis of PKDL and VLR as well in restricting parasite transmission. Noteworthy, we recently showed that PKDL and VLR cases are potential reservoirs for transmission of infection (Mondal et al., 2018). A second generation rK39 rapid test based on validated cutoff titer(s) to identify progression to VL and early detection of VLR and PKDL now appears to be required.

Our findings regarding VL patients at risk for TF are interesting since our data suggest that a low rK39 antibody titer was associated with TF. This could imply a weakened immune response in these patients to the infection due to immune-suppression from a high parasite burden or malnutrition of the host. Though we did not find significant differences between groups regarding their body mass index, the TF group had statistically significantly lower hemoglobin levels compared to VL cured controls indicating a potential micronutrient deficiency. Anti-leishmanial drugs including liposomal amphotericin B have immunostimulatory effects in addition to parasiticidal effects that is dose-dependent (Murray, 2001). Immunosuppression in the TF group could explain why these VL patients failed to respond to a standard single dose of 10 mg/kg, but they did response to a total dose of 15 mg/kg AmBisome divided into three treatments. Additional studies are required to confirm immune-suppression and TF.

The present study has some limitations. One is the small sample size in each group. Nevertheless, it provides very useful information for a new window of opportunity for early detection of TFs, relapses and PKDL based on quantitative measurement of rK39 antibody levels. Furthermore, we used only a serological biomarker, which is dependent on many factors including individual’s immune response, nutrition and genetic factors. Detecting the presence of the parasite including nucleic acids, metabolites and antigens may also represent bona-fide predictors of infection/disease outcome. However, such signature particles from LD parasite are yet to be validated, some of them even for diagnostic purposes. In our previous study we demonstrated the applicability of qPCR as a mean for both detection and quantification of parasite DNA to allow treatment monitoring of VL and PKDL patients (Hossain et al., 2017). Recently, Moulik et al. (2017) reported that parasite kDNA could be used as the promising bio-marker for the prognosis of PKDL patients. Full investigation of parasite kinetics (parasite load) through longitudinal studies is still required to validate this strategy before large scale application.

Another issue is the case definition of “asymptomatic cases” for which there is no clear consensus. Different methods exist to determine *Leishmania* infection status including *Leishmania* specific antibody, antigen, nucleic acid detection or markers of cellular immunity in combination with a clinical assessment of symptoms. According to previous studies, asymptomatically infected persons could be defined as those with no clinical signs or symptoms of VL but are positive in at least one marker of infection including the Leishmanin Skin Test (LST), DAT, rK39 antibody ELISA, IFAT or qualitative/quantitative PCR to detect *Leishmania* DNA (Chakravarty et al., 2019). Most previous studies, including this study, have used the rK39 antibody ELISA/RDT as the biomarker because it has been widely validated as a diagnostic test. Finally, we performed a semi-quantitative ELISA for measuring antibody titer, while a quantitative ELISA could bolster the study findings.

## Conclusion

Quantification of rK39 antibody serum levels could represent an important biomarker for predicting which asymptomatic cases will progress to VL and further could help to predict VL treatment failure, relapse and evolution to PKDL. Validation studies are required through a larger prospective field study. More research involving parasite-specific biomarkers is required to develop better diagnostic and prognostic tools for VL, and facilitate completion of the VL elimination agenda.

## Data Availability Statement

The datasets generated for this study are available on request to the corresponding author.

## Ethics Statement

The studies involving human participants were reviewed and approved by IRB ICDDR, B. Written informed consent was obtained from the participants and for minors, consent was obtained from the parents or legal guardians.

## Author Contributions

DM, PG, CH, PO, JR-P, MD, GM, AK, and DA conceived and designed the study. PG, RC, FH, MK, AA, and RN performed the experiments and maintained the data source. DM, AA, PG, and PO performed the statistical analysis. DM, CH, JR-P, MD, AK, GM, DA, and PO drafted the manuscript. All authors read and approved the final version of the manuscript.

## Disclaimer

The authors alone are responsible for the views expressed in this article and they do not necessarily represent the views, decisions or policies of the institutions with which they are affiliated.

## Conflict of Interest

The authors declare that the research was conducted in the absence of any commercial or financial relationships that could be construed as a potential conflict of interest.
